# Multi-contrast atherosclerosis characterization (MATCH) of carotid plaque with a single 5-min scan: technical development and clinical feasibility

**DOI:** 10.1186/s12968-014-0053-5

**Published:** 2014-07-25

**Authors:** Zhaoyang Fan, Wei Yu, Yibin Xie, Li Dong, Lixin Yang, Zhanhong Wang, Antonio Hernandez Conte, Xiaoming Bi, Jing An, Tianjing Zhang, Gerhard Laub, Prediman Krishan Shah, Zhaoqi Zhang, Debiao Li

**Affiliations:** 1 Biomedical Imaging Research Institute, Cedars-Sinai Medical Center, Los Angeles, CA, USA; 2 Department of Radiology, Anzhen Hospital, Capital Medical University, Beijing, China; 3 Department of Bioengineering, University of California, Los Angeles, CA, USA; 4 Department of Anesthesiology, Cedars-Sinai Medical Center, Los Angeles, CA, USA; 5 MR R&D, Siemens Healthcare, Los Angeles, CA, USA; 6 MR Collaborations NE Asia, Siemens Healthcare, Beijing, China; 7 Oppenheimer Atherosclerosis Research Center, Cedars-Sinai Medical Center, Los Angeles, CA, USA; 8 Atherosclerosis Prevention and Management Center, Cedars-Sinai Medical Center, Los Angeles, CA, USA

**Keywords:** Carotid plaque, Atherosclerosis, Multi-contrast, Composition characterization, Magnetic resonance

## Abstract

**Background:**

Multi-contrast weighted imaging is a commonly used cardiovascular magnetic resonance (CMR) protocol for characterization of carotid plaque composition. However, this approach is limited in several aspects including low slice resolution, long scan time, image mis-registration, and complex image interpretation. In this work, a 3D CMR technique, named Multi-contrast Atherosclerosis Characterization (MATCH), was developed to mitigate the above limitations.

**Methods:**

MATCH employs a 3D spoiled segmented fast low angle shot readout to acquire data with three different contrast weightings in an interleaved fashion. The inherently co-registered image sets, hyper T1-weighting, gray blood, and T2-weighting, are used to detect intra-plaque hemorrhage (IPH), calcification (CA), lipid-rich necrotic core (LRNC), and loose-matrix (LM). The MATCH sequence was optimized by computer simulations and testing on four healthy volunteers and then evaluated in a pilot study of six patients with carotid plaque, using the conventional multi-contrast protocol as a reference.

**Results:**

On MATCH images, the major plaque components were easy to identify. Spatial co-registration between the three image sets with MATCH was particularly helpful for the reviewer to discern co-existent components in an image and appreciate their spatial relation. Based on Cohen’s kappa tests, moderate to excellent agreement in the image-based or artery-based component detection between the two protocols was obtained for LRNC, IPH, CA, and LM, respectively. Compared with the conventional multi-contrast protocol, the MATCH protocol yield significantly higher signal contrast ratio for IPH (3.1 ± 1.3 vs. 0.4 ± 0.3, *p* < 0.001) and CA (1.6 ± 1.5 vs. 0.7 ± 0.6, *p* = 0.012) with respect to the vessel wall.

**Conclusions:**

To the best of our knowledge, the proposed MATCH sequence is the first 3D CMR technique that acquires spatially co-registered multi-contrast image sets in a single scan for characterization of carotid plaque composition. Our pilot clinical study suggests that the MATCH-based protocol may outperform the conventional multi-contrast protocol in several respects. With further technical improvements and large-scale clinical validation, MATCH has the potential to become a CMR method for assessing the risk of plaque disruption in a clinical workup.

## Background

Disrupted carotid atherosclerotic plaques can lead to transient ischemic attack and cerebral thrombo-embolic stroke, significantly contributing to mortality and morbidity worldwide [1]-[3]. Accurate assessment of plaque stability and identification of the lesion at high risk for disruption is of vital importance for improving disease management as well as reducing public health burden. The vulnerability of a plaque is primarily related to its compositional characteristics [4]-[6]. High-resolution cardiovascular magnetic resonance (CMR) is a viable tool for non-invasive characterization of plaque composition [7],[8].

To date, the most commonly used CMR technique for plaque characterization is multi-contrast weighted imaging that involves a series of scans (e.g. T1- and T2-weighted [T1-w, T2-w] black-blood turbo spin-echo [TSE], and bright-blood time-of-flight [TOF]) to differentiate major plaque components, including lipid-rich necrotic core (LRNC), intra-plaque hemorrhage (IPH), calcification (CA), loose matrix (LM), and fibrous tissue [9]-[14]. Despite numerous successes in previous studies, the multi-contrast approach has four major limitations: (a) limited slice resolution associated with two-dimensional (2D) TSE imaging and potential repositioning error when used in serial studies; (b) long acquisition time (approximately 20-30 min); (c) image mis-registration due to inter-scan subject motion; (d) complex image interpretation for differentiating various components based on their signal patterns on multi-contrast weightings. While continued technical improvements and research efforts have been made during the past decade, routine application of CMR for plaque characterization in clinical work-up has not become standard practice [15].

A highly desired CMR technique would be capable of detecting multiple plaque components while mitigating all of the above issues. First, the technique should be based on a three-dimensional (3D) acquisition to reduce partial volume effects and relax the requirements for positioning, both of which are relevant to the accuracy of plaque assessment in tortuous carotid arteries [16],[17]. In fact, 3D imaging has been attempted for assessing carotid wall morphology [18]-[23] or detecting a specific plaque constituent such as IPH [24]-[26], LRNC [27], and CA [28].

Second, the technique should ideally require one scan only that provides multiple image contrasts in an interleaved fashion. This may help shorten the examination time and avoid the mis-registration issue. Multi-echo TSE is perhaps one of the earliest techniques adopting this idea to generate both T2- and proton density-weighted contrasts in carotid plaque imaging. Recent developments on multi-contrast imaging were still limited to offering only two contrasts in a single scan, one for general wall or lumen geometry and the other for one specific plaque component, such as IPH [25] and CA [28].

Third, the multiple image contrasts provided by the technique should be optimized and, if possible, respectively be tailored to one of major components to allow a simplified analysis of composition. Several component-specific contrast weightings have previously been proposed. Heavily T1-weighted contrast created by a nonselective inversion preparation (i.e. MPRAGE), for example, has been shown to be highly sensitive to IPH [24],[26],[29],[30]. Another example is the gray-blood contrast that better discriminates superficial calcific nodules from the carotid wall and lumen irrespective of its hypo-intense signals and juxtaluminal location [28].

In this work, a 3D CMR technique, named Multi-contrast ATherosclerosis CHaracterization (MATCH), was developed to meet the aforementioned needs. The technique features an interleaved acquisition of three image sets with different contrast weightings in a 5-min scan. The first two contrasts, black-blood hyper T1-w and gray-blood, are used to identify IPH and CA, respectively. A third T2-w contrast, in addition to providing overall plaque morphology, can detect LM and LRNC and differentiate acute and recent hemorrhage when combined with the hyper T1-w contrast. The MATCH sequence was optimized for 3.0-Tesla (3 T) based on computer simulations and testing on healthy volunteers and then evaluated in a pilot study of patients with carotid plaque, using the conventional multi-contrast protocol as a reference.

## Methods

### Sequence design

**Figure 1:**
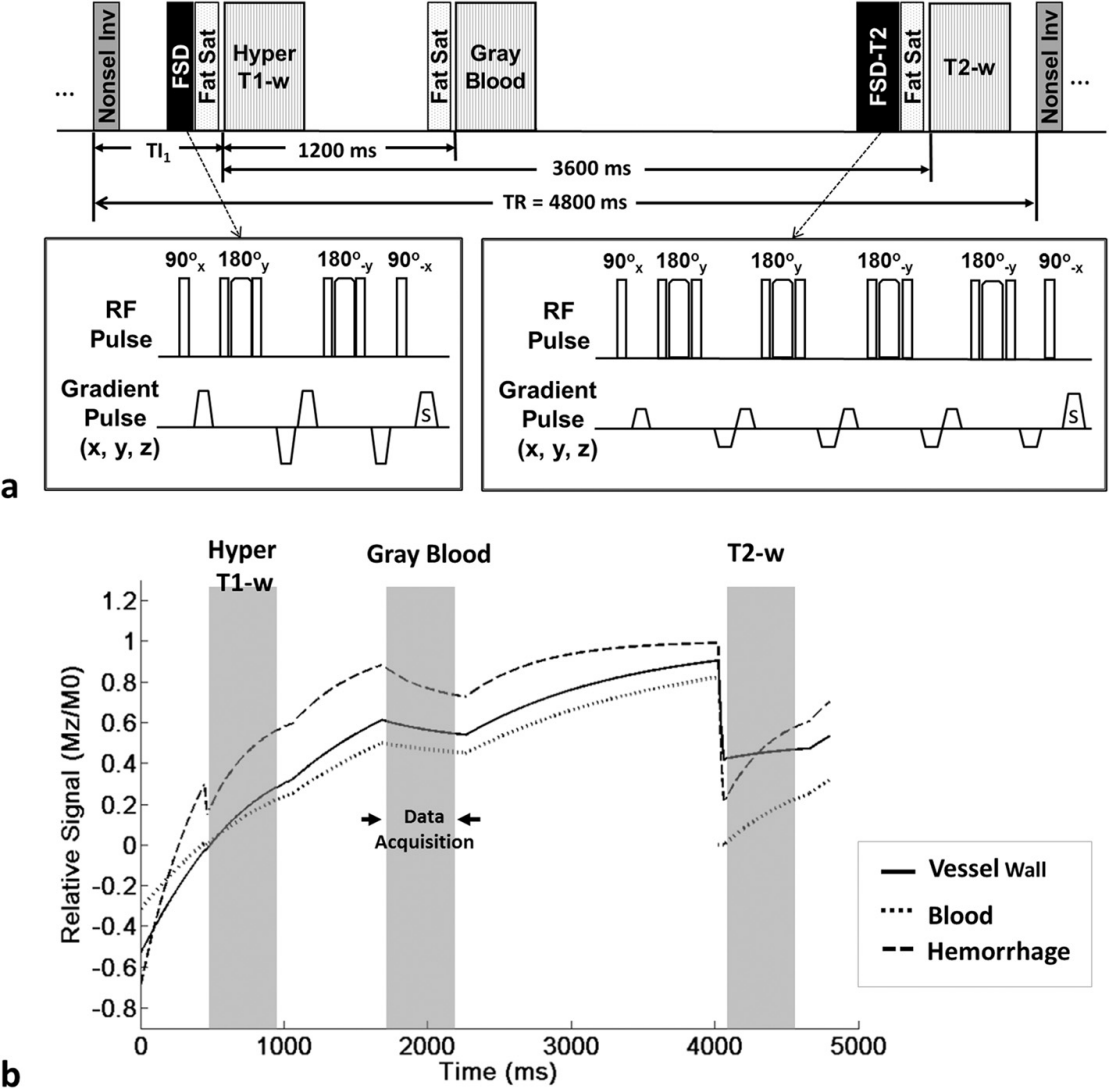
**Schematic diagram (a) and signal simulations (b) of the MATCH sequence. a**. Three contrast weightings following a nonselective inversion (Nonsel Inv) pulse and various inversion-recovery times (TIs) are acquired during each TR (4800 ms). At the first TI, TI_1_, hyper T1 weighting is created to suppress the signals from all non-hemorrhagic vessel wall tissues while highlighting IPH because of its shorter T1. Flow-sensitive dephasing (FSD) preparation is applied before data acquisition to suppress luminal blood signals. At the second TI (TI_1_ + 1200 ms), blood and non-hemorrhagic vessel wall tissues will both recover moderately to create neutral weighting or gray-blood images to highlight the dark CA. At the third TI (TI_1_ + 3600 ms), all tissues have largely recovered and black-blood T2-w images are acquired with a combined T2 and FSD preparation. The acquisition of each contrast weighting is based on a low-flip-angle FLASH readout that is immediately after a chemically selective fat saturation module (Fat Sat). The two FSD preparative modules consist of 2 and 4 composite refocusing pulses, respectively, to minimize (17-ms-long) or strengthen (40-ms-long) T2-decay effects while maintaining homogenous B_1_ field. **b**. With the TI_1_ optimized, steady-state signal evolutions for the normal vessel wall, IPH, and arterial blood during a TR period were simulated. The arterial blood signal involution shown herein is based on the assumption that inflow fresh blood is ignored.

The proposed technique employs a 3D spoiled segmented fast low angle shot (FLASH) readout to acquire data with three different contrast weightings following a nonselective inversion pulse and various inversion-recovery times (TIs) (Figure 1a).

At the first TI, hyper T1 weighting is created to suppress the signals from all non-hemorrhagic vessel wall tissues while highlighting IPH because of its shorter T1. Flow-sensitive dephasing (FSD) preparation is applied before data acquisition to suppress luminal blood signals [31]-[33]. At the second TI, blood and non-hemorrhagic vessel wall tissues will both recover moderately to create neutral weighting or gray-blood images to highlight the dark CA. At the third TI, all tissues have largely recovered and black-blood T2-w images are acquired with a combined T2 and FSD preparation. T2-weighted images are used to characterize LM and LRNC and facilitate differentiation between acute and recent IPH. The criteria for resolving plaque components based on the three sets of images acquired using the MATCH sequence are summarized in Table 1.

**Table 1:** Criteria for determining plaque components from the MATCH imaging protocol based on components’ signal intensity relative to adjacent sternocleidomastoid muscle

	**MATCH contrast weighting**		
**Component**	**Hyper T1-w**	**Gray blood**	**T2-w**
IPH (along with LRNC)	+		
CA		-	
LM	=		+
LRNC without IPH	=		-

IPH: intra-plaque hemorrhage; CA: calcification; LM: loose matrix; LRNC: lipid-rich necrotic core. +: hyper-intense; =: iso-intense; -: hypo-intense.

### Optimization

A hyperbolic secant adiabatic inversion pulse was used for uniform inversion at 3 T. The FSD module in the hyper T1-w acquisition had two composite (90°_x_-180°_y_-90°_x_) refocusing pulses weighted in an MLEV pattern to ensure second-order corrections to B_1_-field inhomogeneity [34]. A cumulative first-order gradient moment (m_1_) of 990 mT∙ms^2^/m, which has been suggested to be adequate for carotid flow suppression given the voxel dimension used in this work [32], was chosen to allow a relatively short preparation time of 17 ms to alleviate the T2 weighting. The second FSD module was 40 ms long to generate T2 weighting. To further improve the insensitivity to B_1_-field inhomogeneity, four composite refocusing pulses were used [34].

A low flip angle of 8° was used in the FLASH readout for minimal interruption of the T1-recovery of vessel wall magnetizations and reducing the incidence of image artifact due to a strong k-space signal modulation. Chemically selective fat saturation was applied immediately before each readout train to improve outer wall boundary definition. To preserve the effects of the FSD preparation and fat saturation, centric phase-encoding was employed in readouts.

A TR of 4800 ms was chosen as a trade-off between the total scan time and the need of near full T1 recovery prior to the T2-w acquisition. In this context, the first TI, denoted as TI_1_, was optimized through computer simulations based on the Bloch equations in MATLAB (R2009b, Mathworks, Natick, MA); TI_1_ should approximately null the signals from all non-hemorrhagic vessel wall tissue and IPH appears as a “hot spot” in images. The second and third TIs were shifted by 1200 ms (for moderate T1 recovery) and 3600 ms (for near full T1 recovery), respectively, relative to TI_1_. The simulated tissues included arterial wall media (T1/T2 = 1115/50 ms), arterial blood (1550/275 ms), IPH (500/25 ms) [26],[35]. Fibrotic tissue (T1 = 1000 ms) [28], a major intra-plaque occupant, was also considered in the optimization of TI_1_. The sequence parameters used in simulations were the same as in *in-vivo* scans (Table 2).

**Table 2:** Relevant imaging parameters for the sequences used

	**TOF**	**T1-w TSE**	**T2-w TSE**	**MATCH**
TE/TR (ms)	3.6/21	12.0/800	59.0/4000	4.2/10.8
Field-of View (mm^2^)	150 × 200	140/160 × 140/160	140/160 × 140/160	140/160 × 140/160
In-plane resolution (mm^2^)	0.87 × 0.52	0.55/0.63 × 0.55/0.63	0.55/0.63 × 0.55/0.63	0.55/0.63 × 0.55/0.63
Number of slices	84 slices (with 3 slabs)	16	16	16 (plus 4-slice oversampling)
Slice thickness (mm)	1.0 (with 19% overlap)	2	2	2
Number of Average	1	2	2	1
Flip angle (^o^)	25	136-170	120-160	8
Echo train length	-	7	12	55-67*
Bandwidth (MHz)	250	407	407	130
Parallel factor (GRAPPA)	2	-	-	2
Scan time (min:sec)	2:27	2:56	3:24	4:45

*The # of segments varied with oversampling in the phase-encoding direction which ranged from 20-30%.

To verify the simulation results and characterize general image contrast, the sequence was tested on four healthy subjects. A 3 T whole-body system (MAGNETOM Verio; Siemens AG, Erlangen, Germany) and a 4-channel carotid coil (Machnet BV, Roden, The Netherlands) were used for data acquisitions.

### Patient studies

Six male patients (aged 56-77, mean age = 67) with ultrasonography-documented carotid artery stenosis (Table 3) were recruited in a feasibility study using a 3 T whole-body system (MAGNETOM Verio; Siemens AG, Erlangen, Germany) and an 8-channel carotid coil (Shanghai Chenguang Medical Technologies, Shanghai, China). After obtaining informed consent from each patient, MATCH imaging was conducted during their scheduled clinical CMR examination that included the conventional multi-contrast (black-blood multi-slices 2D T1-w and T2-w TSE with saturation bands, multi-slab 3D TOF) imaging protocol [11]. All these scans were performed axially with the same imaging volume centered at the bilateral bifurcations. The slice thickness (2 mm) and in-plane spatial resolution of MATCH matched those of TSE. TOF had different slice thickness and in-plane spatial resolution as a part of the clinical protocol.

**Table 3:** Artery-based composition analyses: MATCH vs. Conventional protocol

**Artery**		**LRNC**		**IPH**		**CA**		**LM**	
	**Stenosis by Ultrasound (%)**	**MATCH**	**Conv.**	**MATCH**	**Conv.**	**MATCH**	**Conv.**	**MATCH**	**Conv.**
P1.R	50%	+	+	+	+	+	+	+	+
P2.R	75%	+	+	+	+	+	+	+	+
P2.L	50%	-	-	-	-	+	+	+	+
P3.R	70%	+ (-)*	+ (-)	+	+	-	-	-	+
P3.L	75%	+ (-)	+ (-)	+	+	+	+	+	+
P4.R	50%	+	+	-	-	+	-	+	+
P4.L	50%	+	+	+	+	+	+	+	+
P5.L	75%	-	-	-	-	+	+	-	-
P6.R	50%	+	+	-	-	+	-	+	+
P6.L	50%	+	+	-	-	+	+	+	+
Kappa (p-value)		1.0 (0.002)		1.0 (0.002)		0.41 (0.107)		0.62 (0.035)	

LRNC: lipid-rich necrotic core; IPH: intra-plaque hemorrhage; CA: calcification; LM: loose matrix. Conv.: Conventional protocol. +: detected; -: undetected. *The symbol in the parenthesis denotes the results when only IPH-free slices were read.

### Image analysis

All image data sets were processed on a workstation (Leonardo; Siemens AG, Erlangen, Germany). T2-w MATCH images served as a reference of arterial wall anatomy for determining plaque composition using the other two sets of images of MATCH.

In healthy subjects, for each of MATCH contrast weightings, the center 5 slices of each artery were chosen for signal measurement. On every slice, two regions-of-interest (ROI) were manually drawn to respectively outline the arterial lumen and wall for measuring their signal intensity S_l_ and S_w_. Noise level (σ_n_) was measured as the standard deviation of signals from an ROI (~100 mm^2^) manually drawn in an artifact-free air region that was near the artery. The signal-to-noise ratio (SNR) of the wall and lumen (calculated as S_l (w)_/σ_n_) as well as the wall-lumen contrast-to-noise ratio (CNR) (calculated as |S_w_-S_l_|/σ_n_) were calculated for each slice and averaged over all 5 slices. The above ROIs were first prescribed on the T2-w images and then copied to the other two contrast weightings to ensure consistency of measurement locations. Due to the use of parallel imaging, absolute SNR and CNR were difficult to quantify. Instead, the values calculated herein were counted as apparent SNR and apparent CNR, respectively. They were aimed to help appreciate the relative image contrasts of different MATCH image sets and reveal whether they were in accord with the theoretical sequence design.

For patient studies, the images from each artery underwent the location matching (including image reformation in 3D TOF) process to account for the inconsistency in slice number and thickness between the two protocols and inter-scan motion. The images that had all four spatially registered scans were further screened for diagnostic quality (i.e. overall image quality, vessel wall clarity). Diagnostic images were finally included in subsequent analyses.

Blinded image review for composition identification was performed by a radiologist (with 9-year experience in carotid plaque MR characterization) with the two imaging protocols separated by two weeks. The presence of IPH, CA, LRNC, and LM were determined using the criteria summarized in Table 1 for the MATCH protocol and those in a recent review article for the conventional protocol [36]. In addition, for both protocols, the age of each identified IPH, i.e. acute or recent, was recorded according to its signal intensity relative to adjacent sternocleidomastoid muscle on T2-w images: iso-intensity or hypo-intensity for the acute type and hyper-intensity for the recent type [37]. Artery-based and image-based agreements in the detection of individual components by the two protocols were respectively determined using a Cohen’s kappa test. According to Landis and Koch [38], the agreement was rated as follows: kappa 0 to 0.2 indicated slight agreement, 0.21 to 0.4 fair agreement, 0.41 to 0.60 moderate agreement, 0.61 to 0.8 substantial agreement, and 0.81 upward excellent agreement. Note that the plaque with detected IPH was counted as a plaque with LRNC during review, and LRNCs underwent agreement analysis for both scenarios – IPH present and IPH absent.

The design of hyper T1-w and gray-blood contrasts is relatively unique in MATCH, aiming for better discerning IPH and CA, respectively. To unravel such, the contrast ratio (CR) between the component and the regular vessel wall was calculated as [S_1_-S_2_]/S_2_ and compared between the two protocols using a paired Student’s *t* test. More specifically, the signal was measured in all images where IPH or CA was identified and clearly demarcated in both protocols; for simplicity, IPH (S_1_) and the vessel wall (S_2_) was analyzed on T1-w TSE and hyper T1-w MATCH, whereas CA (S_2_) and the vessel wall (S_1_) on TOF and gray-blood MATCH since these contrast weightings were the most relevant for the discrimination of IPH or superficial calcification. Window level adjustment was performed on each of images to ensure optimal contrast between the vessel wall and lumen. The component of interest was then manually outlined along its boundary that was visually determined by the reviewer (with 7-year carotid wall MRI) based on signal contrast (hypo- or hyper-intense). SNR or CNR was not measured herein because parallel imaging was used in the MATCH protocol.

All the above statistical tests were performed using SPSS (version 16.0; SPSS Inc., Chicago, IL). Statistical significance was defined at the *p* < 0.05 level. Data are presented as means ± standard deviations.

## Results

### Computer simulations and *in vivo* verification

**Figure 2:**
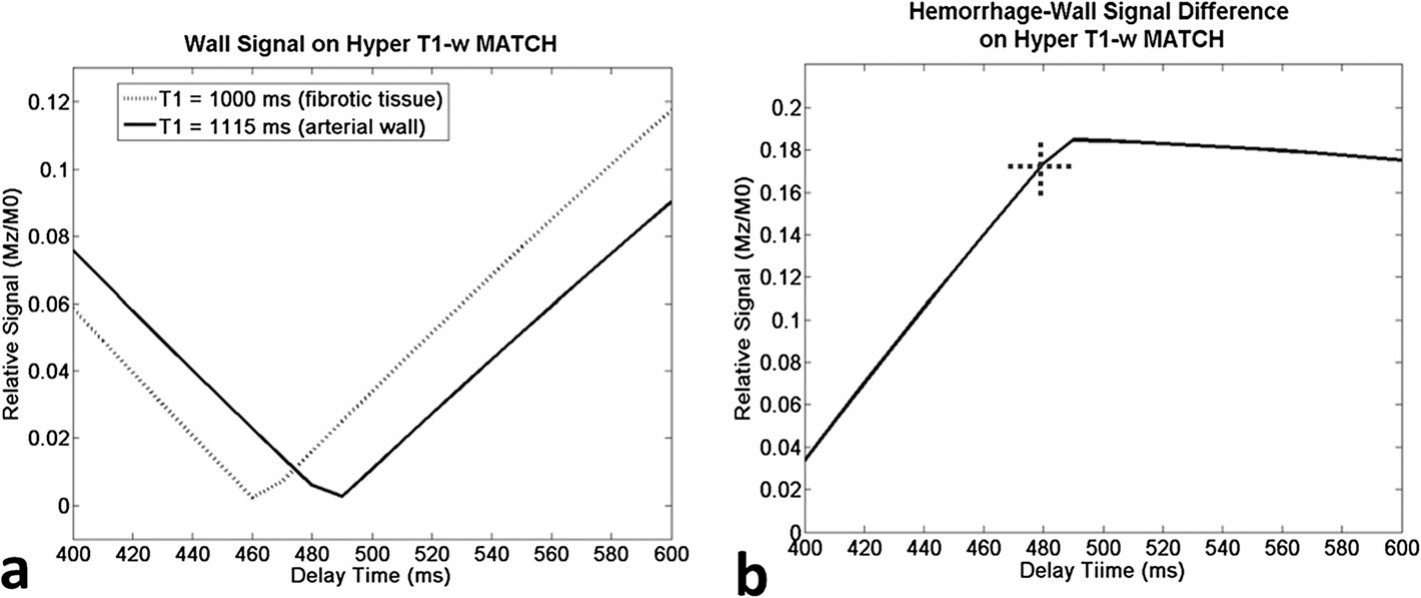
**Computer simulations for the optimization of the first inversion-recovery time (TI).** A TI of 490 ms can approximately null the signals of the “normal” arterial wall, but the fibrotic tissue required a shorter TI time, i.e. 460 ms **(a)**. As a trade-off, 480 ms was selected as the optimal delay time, providing a difference of 0.173 in normalized signals between the wall and IPH on the hyper T1-w contrast (**b** cross hair).

For the interrogated range of 400 to 600 ms, an optimal TI (490 ms) existed that could approximately null the signals of the “normal” arterial wall and provided a maximal difference of 0.185 in normalized signals between the wall and IPH on the hyper T1-w contrast (Figure 2a, b). However, the largely distributed fibrotic tissue required a shorter TI, 460 ms. As a trade-off, 480 ms was selected as the optimal TI_1_, providing a difference of 0.173 in normalized signals between the wall and IPH on the hyper T1-w contrast (Figure 2b cross hair). With the choice, steady-state signal evolutions (after 10 iterations) for the normal vessel wall, IPH, and arterial blood during a TR period were simulated and shown in Figure 1b.

**Figure 3:**
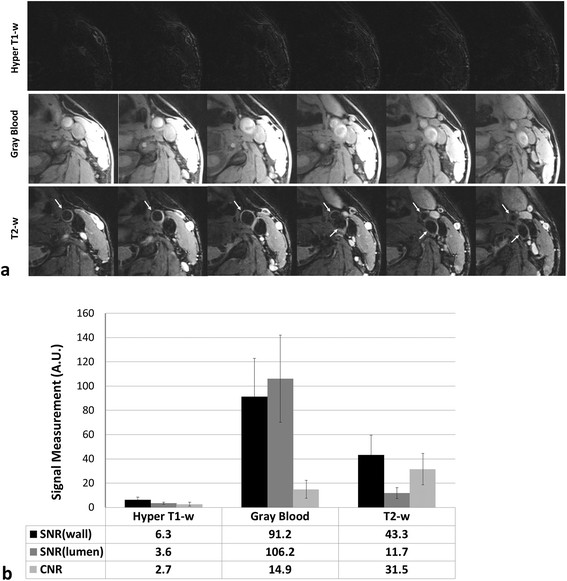
**Healthy subject example images (a. 6 contiguous slices around the left carotid bifurcation) and signal measurements from 8 carotid arteries (b) from the MATCH imaging protocol.** All three contrast weightings are inherently registered. Note that blood attenuation is sufficient in both hyper T1-w and T2-w images. A.U.: arbitrary unit. The error bars in b denotes a standard deviation of the specific quantity.

Good image quality was observed in MATCH images of all 4 healthy subjects (Figure 3a). The three image sets were intrinsically co-registered in spatial location. The normal vessel wall and lumen were both substantially attenuated on hyper T1-w images using the optimized delay time. They were of moderate signal intensity on gray blood images with the lumen appearing a little brighter, presumably due to the inflow effect. On T2-w images, the carotid wall was well depicted with the lumen and epivascular fat sufficiently attenuated. The above image contrast was also corroborated by the signal measurements from the 8 healthy carotid arteries (Figure 3b).

### Patient studies

The two protocols were successfully performed in all 6 patients. The scan time was 12-15 min for the conventional protocol (including sequence set up and B_0_ field shimming) and 5 min for the MATCH protocol. During the studies, patients were instructed to remain still throughout the imaging session. No evident motion artifacts were observed within MATCH and each of the conventional sequences. All three MATCH contrasts were spatially co-registered. However, slight mis-registration was observed between T1-w TSE and T2-w TSE in one subject or between TOF and TSE in two subjects. We stipulate that the patient could remain still well in a scan due to the prior instruction and reminder right before the scan, but more likely he/she moved the head during the gap between scans. For a total of 192 artery images (6 patients × 16 slices × 2 arteries), 32 arterial images (from two arteries) were excluded from analyses because of the presence of stenting and surgical removal of intima, respectively, 7 excluded due to spatial mismatch between the two protocols, and 17 excluded due to poor diagnostic quality (11, incomplete wall structure due to low signals on both MATCH and TSE/TOF images; 4, incomplete wall structure due to low signals on MATCH; 2, flow artifacts on MATCH). The remaining 136 images were subjected to analyses.

**Figure 4:**
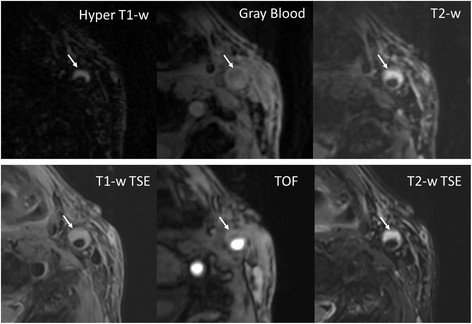
**(Patient 3) For the recent intra-plaque hemorrhage, hyper-intense contrast is its characteristic appearance on hyper T1-w and T2-w MATCH.** The component is also verified on conventional T1-w (hyper-intense), T2-w (hyper-intense) TSE and TOF (hyper-intense). Notably, the contrast of the hemorrhage in this case is much sharper on hyper T1-w MATCH than on T1-w TSE.

**Figure 5:**
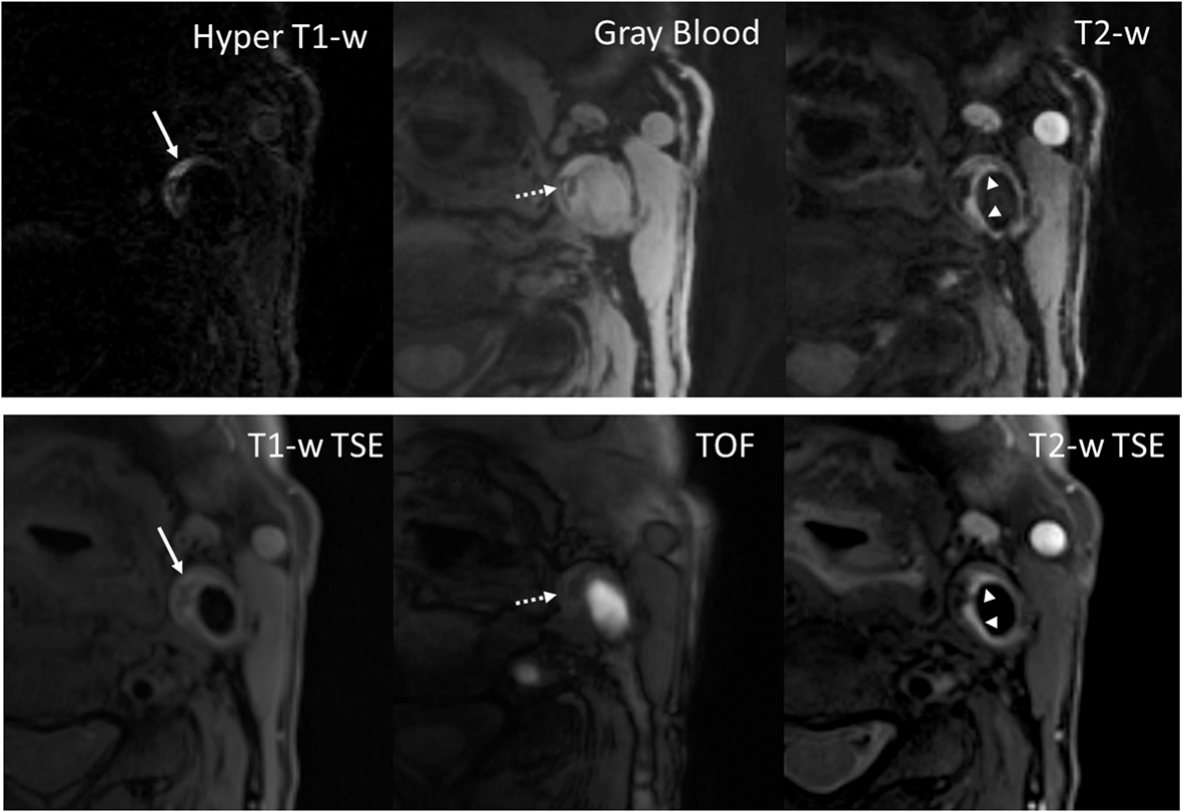
**(Patient 4) A slice with co-existent plaque components including acute intra-plaque hemorrhage (solid arrows), calcification (dashed arrows), and loose matrix (arrowheads).** With the MATCH protocol, the unique contrast weightings and spatial coregistration facilitate easier identification of co-existent components and better appreciation of their spatial relations. The loose matrix is also hyper-intense on T1-w TSE, mimicking hemorrhage. However, it is not as hyper-intense as hemorrhage on TOF.

**Figure 6:**
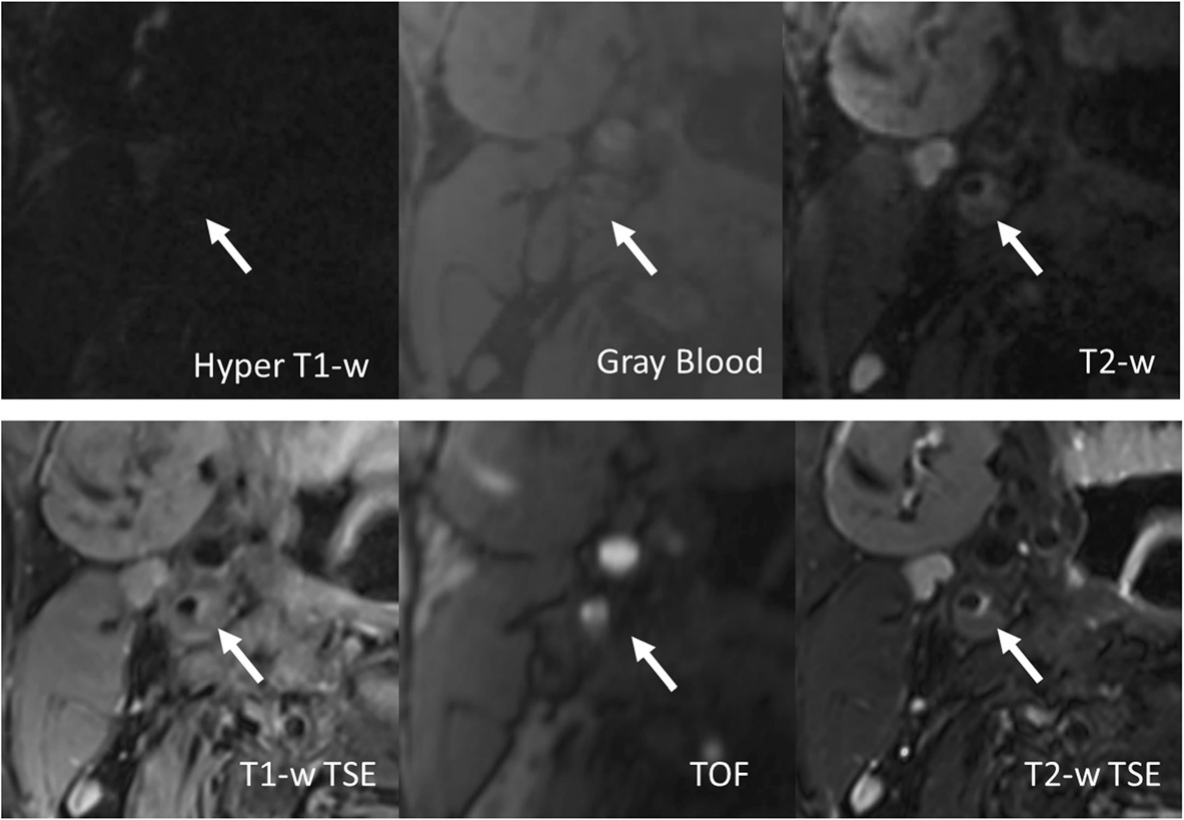
**(Patient 2) A lipid-rich necrotic core without hemorrhage appears hypo-intense on both T2-w TSE and T2-w MATCH.** Notice that there is signal drop in the lumen on the gray blood image due to flow signal dephasing associated with fast flow at the high degree stenosis.

The remaining 10 arteries all have at least one of 4 major components. On MATCH images, the major components were easy to identify by the reader. IPH (Figures 4 and 5, arrows) appeared hyper-intense on hyper T1-w MATCH images, but hyper-intense (recent IPH) or iso-intense (acute IPH) on T2-w MATCH images. CA (Figure 5, dashed arrow) appeared as focal signal voids on gray-blood MTCH images. LM (Figure 5, arrowheads) appeared hyper-intense on T2-w MATCH but not on hyper T1-w MATCH images. LRNC was depicted as a hypo-intense region on T2-w MATCH images when there was no IPH in it (Figure 6). Spatial co-registration between the three image sets with MATCH was particularly helpful for the reviewer to discern co-existent components in an image and appreciate their spatial relation, as exemplified by the case shown in Figure 5. For the artery-based component detection, excellent agreement was obtained for LRNC (regardless of the presence of IPH) (κ = 1.0) and IPH (κ = 1.0), good agreement obtained for LM (κ = 0.62), and moderate agreement obtained for CA (κ = 0.41) (Table 3). For the image-based component detection between the two protocols, excellent agreement was obtained for LRNC (including images with IPH detected) (κ = 0.89) and IPH (κ = 0.83), and substantial agreement obtained for CA (κ = 0.70) and LM (κ = 0.78) (Table 4). Furthermore, IPH-free LRNCs received slice-based good agreement (κ = 0.86; MATCH: detected in 20 out of 107 images; conventional protocol: detected in 23 out of 107 images; 19 images in common) between the two protocols as well. In the 22 images with IPH detected by both protocols, a complete consensus in the classification of IPH (22 images with IPH in common) into the acute (6 images) and recent (16 images) types was achieved.

**Table 4:** Slice-based composition analyses: MATCH vs. Conventional protocol

		**Conventional Protocol (T1-w TSE, T2-w TSE, TOF)**							
		**LRNC**		**IPH**		**CA**		**LM**	
		+	-	+	-	+	-	+	-
MATCH Protocol	+	46	3	22	7	31	16	23	2
	-	4	83	0	107	1	88	8	103
# of slice in agreement (%)		129 (94.9%)		129 (94.9%)		119 (87.5%)		126 (92.6%)	
Kappa (p-value)		0.89 (<0.001)		0.83 (<0.001)		0.70 (<0.001)		0.78 (<0.001)	

LRNC: lipid-rich necrotic core; IPH: intra-plaque hemorrhage; CA: calcification; LM: loose matrix. +: detected; -: undetected.

Note: the plaque with IPH detected was also counted as a plaque with LRNC.

**Figure 7:**
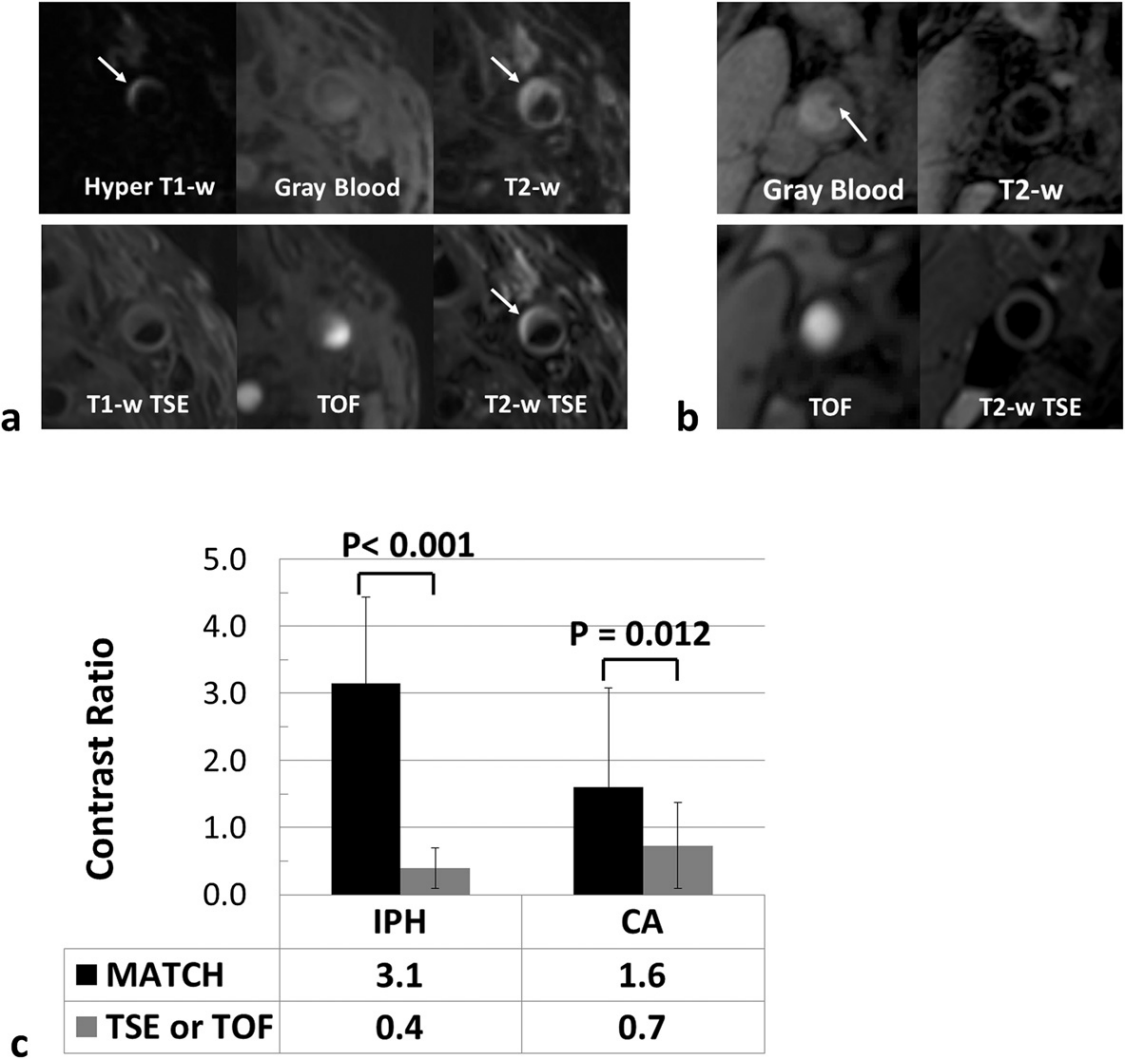
**MATCH sequence yielded more detection of hemorrhage-like and calcification-like locations. a**. (Patient 3) The recent hemorrhage (arrows) indicated by MATCH is hyper-intense on T2-w TSE but not on T1-w TSE; thus it is counted as loose matrix by the conventional protocol. **b**. (Patient 7) A superficial calcified nodule is clearly depicted on gray blood MATCH. Such a component, when protruding into the arterial lumen, is usually not well observable with the conventional multi-contrast protocol. **c**. The contrast ratios between the hemorrhage/calcification and the regular arterial wall are significantly higher (p < 0.001 and p = 0.012, respectively) with the MATCH protocol.

The MATCH protocol yielded more incidence of IPH (Figure 7a) and CA (Figure 7b) compared with the conventional protocol. All IPH-like and all but one CA-like locations as detected by the conventional protocol were also identified on MATCH images; however, the MATCH protocol indicated 7 additional “IPH” (categorized as the recent type) and 15 additional “CA” (Table 3). Five locations appeared hyper-intense on both hyper T1-w and T2-w MATCH images, but only on T2-w TSE, and therefore were counted as LM by the conventional protocol (Figure 7 a, arrows). Both of the two components had significantly higher signal contrast (based on 22 images for IPH: CR = 3.1 ± 1.3 vs. 0.4 ± 0.3, *p* < 0.001; 23 images for CA: CR = 1.6 ± 1.5 vs. 0.7 ± 0.6, *p* = 0.012) with respect to the vessel wall on MATCH images, making their detection remarkably straightforward (Figure 7b).

## Discussion

To the best of our knowledge, the proposed MATCH sequence is the first 3D CMR technique that acquires spatially co-registered multi-contrast image sets in a single scan for adequate characterization of carotid plaques. Compared with the conventional multi-contrast protocol, the presented MATCH CMR protocol features substantially shortened scan time and simplified image interpretation. The pilot study on patients demonstrated that the MATCH protocol is in good agreement with the conventional protocol in the detection of IPH, CA, LM, and LRNC as well as differentiation of IPH age.

To enable a short scan time and expedited identification of these components, the MATCH protocol included a minimum of three contrast weightings. The first contrast weighting, hyper T1-w, adopted a nonselective inversion preparation that has been employed in the MPRAGE method and proven to outperform several other T1-w sequences for the detection of IPH [29]. Such an approach, however, may not adequately suppress the blood signals while achieving good contrast between the IPH and vessel wall. Although a phase-sensitive (PS) reconstruction method could be used to address the issue [25],[39], an FSD black-blood preparation module was used instead to eliminate the need of post-processing and avoid phase manipulation-related errors. With such a combination of magnetization preparations, computer simulations revealed an IPH-wall signal contrast of 0.173 that is substantially higher than that theoretically available from regular MPRAGE imaging [39]. In our pilot study, 7 images with IPH-like signal characteristics were observed by the MATCH protocol only, suggesting its higher sensitivity to IPH than the conventional multi-contrast protocol that included T1-w TSE instead of MPRAGE and thus could be of limited performance according to Ota *et al*. [29].

The second contrast weighting, gray blood, was designed specifically for the detection of CA, especially the superficial calcified nodules. Due to hypointense signals and juxtaluminal location, superficial calcified nodules are often poorly visualized on the black-blood images and likely mistaken as the lumen or wall surface ulceration. On the other hand, the shadowing effect from the bright lumen on the TOF images may limit the discrimination of this constituent from others. In contrast, gray blood contrast makes the lumen slightly brighter than calcification while maintaining an adequate contrast between the lumen and the vessel wall. In contrast to the original implementation by Koktzoglou I. [28], gray blood contrast in this work was acquired following relatively long blood T1-recovery, resulting in a slightly brighter lumen. Nevertheless, the advantages associated with the gray blood contrast were preserved and have been clearly demonstrated in the patient studies.

The third contrast weighting, T2-w, was chosen to provide overall plaque morphology, detect LM and LRNC, and, when combined with the hyper T1-w contrast, differentiate acute and recent hemorrhage. With the optimized parameters, the T2-w MATCH images were nearly identical to the conventional T2-w TSE images with respect to overall image contrast. Therefore, concordant composition analyses were obtained with both characterization protocols in our study.

An interleaved acquisition of the three contrast weightings is one of noteworthy features in MATCH. This may significantly reduce the likelihood of mis-registration between multiple contrasts and potentially avoid substantial data exclusion in patient studies [11],[14]. Easier identification of co-existent components and better appreciation of their spatial relations were perceived by the reviewer when using MATCH. As an important ingredient for the interleaved acquisition strategy, a low-flip-angle segmented FLASH readout employed in this work allowed for a large amount of k-space lines to be acquired and three-phase signal sampling per TR with a very minimal interruption of T1-recovery.

Long scan times commonly associated with 3D imaging for plaque characterization is a limitation because some patients, particularly the elderly in whom carotid atherosclerosis is common, may not tolerate the long procedure. In a recent multi-center trial using the conventional plaque characterization protocol, patient movement-induced motion artifact accounted for 46% of failed cases (15% of all) [40]. Thus, the proposed MATCH protocol was developed so that imaging could be completed within a clinically acceptable scan time, i.e. 5 minutes. The use of fast FLASH readout, interleaved image acquisition, and parallel imaging has contributed to the achievement of this goal. As a trade-off, however, high slice resolution (e.g. 1 mm) or even isotropic spatial resolution was not attempted in this work. Therefore, the reduced partial volume effect by 3D imaging was essentially not demonstrated herein. Nevertheless, the MATCH sequence has the potential to yield higher spatial resolution images if advanced fast imaging methods such as compressed sensing can be integrated into it.

Due to the interleaved acquisition manner and relatively long scan times, MATCH could be more prone to motion than the conventional sequences. Random motion events, such as bulk motion or swallowing, would corrupt all three MATCH data sets and necessitate a repeat scan. In contrast, the conventional protocol may still have part of scans providing valid information. Although no evident motion artifacts were observed in MATCH with the limited patient size in this work, this shortcoming merits a systematic investigation and further technical improvement such as motion self-gating [41].

It should be acknowledged that MATCH is not capable of identifying all important features in carotid atherosclerotic plaques. Thinning/rupture of the fibrous cap has been shown to have the highest hazard ratio compared with IPH and LRNC and therefore serves as one of important imaging marker for plaque vulnerability [42]. However, the current non-contrast MRI techniques have been shown to be poor in reproducibility of identifying fibrous cap [43]. Hence, MATCH, as a non-contrast technique, was not designed in this first development work to characterize the fibrous status. Further innovation is desirable to incorporate the ability into MATCH. Alternatively, contrast-enhanced MRI that has proven good reproducibility of fibrous cap status assessment [44] may be combined with MATCH.

There are several limitations in this study. First, plaque histology, the gold standard for plaque characterization, was not available in the pilot study. The capability of MATCH in detecting each of histological components was evaluated through a comparison with the conventional multi-contrast protocol. Despite good agreement in image-based composition analysis, there was some discrepancy between the two protocols. Histological verification is needed to determine the accuracy of composition analysis. Second, the patient sample size is rather small. This preliminary work presents technical aspects and the feasibility of the new sequence. While the pilot data have been encouraging, a large-scale investigation is warranted to further elucidate its clinical utility. Third, the conventional multi-contrast protocol that served as a reference could be further refined. For example, the TOF sequence should have used the same slice thickness and in-plane resolution as in other scans; MPRAGE [29] and contrast-enhanced T1-w TSE [45]-[47] might be included for an improved detection of IPH and lipid core, respectively, although this could be unsuitable for patients who cannot tolerate longer imaging protocol or have renal insufficiency. Last, some potential technical advantages of the MATCH sequence over the conventional protocol await more stringent validation. For example, higher slice resolution was not demonstrated due to the consideration of scan time and patient tolerance; more simplified image interpretation was only subjectively reported by the image reader but not quantitatively validated by a comparison of interpretation times. Further investigations with focus on these aspects are highly necessary.

## Conclusions

A 3D CMR technique, MATCH, is presented that requires one scan only to concurrently collect three spatially co-registered multi-contrast image sets for comprehensive characterization of carotid plaque composition. Our pilot patient study demonstrates that the MATCH-based protocol has a comparable ability to detect carotid plaque components such as LRNC, IPH, CA, and LM in less time with no problems of mis-registration compared with the conventional multi-contrast protocol. Further technical improvements to reduce scan time and increase slice resolution (better than the current implementation of 2-mm thick slice) are needed to make the technique more useful in the assessment of carotid plaques. Large scale clinical validation, in particular with histology as reference, is warranted to elucidate whether MATCH has the potential to become a CMR method for assessing the risk of plaque disruption in a clinical workup.

## Competing interests

The authors declare that they have no competing interests.

## Authors’ contributions

ZF designed and implemented the sequence, performed numerical simulations and in-vivo validation on healthy volunteers, and analyzed all data and prepared manuscript. WY was in charge of recruiting clinical patients, performing clinical studies, and helped analyzing data. YX participated in the sequence implementation and data acquisition on healthy volunteers. LD and LY participated in the coordination, data acquisition, and data analysis for clinical studies. ZW participated in the data acquisition of clinical studies. AHC participated in design and coordination of healthy volunteer studies. XB and GL helped with the sequence design and implementation. JA and TZ helped with coordination of clinical studies. PKS and ZZ participated in the design of the clinical studies. DL conceived of the sequence design and provided supervision of the whole project and critical review of the manuscript. All authors read and approved the final manuscript.
